# Mobility assessment using wearable technology in patients with late-onset Pompe disease

**DOI:** 10.1038/s41746-019-0143-8

**Published:** 2019-07-22

**Authors:** Alaa Hamed, Christopher Curran, Chad Gwaltney, Pronabesh DasMahapatra

**Affiliations:** 1 Sanofi Genzyme, Cambridge, MA USA; 2 PatientsLikeMe, Cambridge, MA USA; 3 Gwaltney Consulting, Westerly, RI USA

**Keywords:** Outcomes research, Physiology

## Abstract

Late-onset Pompe disease (LOPD) is a rare genetic disorder due to the absence or deficiency of acid alpha-glucosidase enzyme resulting in slowly progressing reduction of muscle strength, causing difficulties with mobility and respiration. Wearable technologies offer novel options to evaluate mobility in a real-world setting. LOPD patients self-reporting LOPD, ≥18 years, US residents, walking (with or without aid), and not on invasive ventilation were recruited for a 6- to 8-week wearable study via patient organizations. Eligible patients were shipped a wearable tracker (Fitbit One™) and completed self-assessment questionnaires. Mobility outcome measures were median step count and peak 1-min activity. In the analyses cohort (*N* = 29), engagement in data sharing was high (94% of patients uploaded data for more than half the study days). Mean age was 43 years, 90% were females, and 93% were diagnosed in adulthood. Mean delay in diagnosis was 10 years; most had disease onset for ≥10 years (55%); some required walking aid (17%) and breathing assistance (38%). Mean step count differed by age (20–39 years: 4071 vs. 40–69 years: 2394, *p* < 0.01), diagnostic delay (<10 years: 3584 vs. ≥10 years: 2232, *p* < 0.05), disease duration (<10 years: 4219 vs. ≥10 years: 2462, *p* < 0.05), and ambulatory status (aided: 1883 vs. unaided: 3408, *p* < 0.05). Patient-reported “fatigue and pain” score was inversely correlated with step count (Pearson’s *r* = −0.42, *p* < 0.05) and peak 1-min activity (Pearson’s *r* = −0.49, *p* < 0.01). This study illustrates a new approach to measure mobility in LOPD patients and establishes a framework for future outcomes data collection.

## Introduction

Pompe disease (acid maltase deficiency disease) is a rare and progressive neuromuscular disorder caused by the absence or deficiency of acid alpha-glucosidase (GAA), the enzyme required for the breakdown of glycogen.^1^ Glycogen accumulation in muscle cell lysosomes results in a variety of symptoms due to potentially fatal myopathy.^1^ While the clinical presentation of Pompe disease is heterogeneous, it is generally classified as either infantile-onset Pompe disease (IOPD) or late-onset Pompe disease (LOPD). In LOPD, symptoms appear ≥1 year of age and patients experience slowly progressive limb-girdle muscle weakness and respiratory insufficiency.^2,3^

Age of onset varies widely in LOPD, with some individuals first experiencing symptoms in early childhood and others in their 60s or 70s^4–7^ The first symptom noted in almost all patients is skeletal muscle weakness, as demonstrated by difficulty running, performing sports, climbing stairs, walking, rising from an armchair, or rising from a lying position.^3,7^ Advancing weakness of the proximal and paraspinal muscles eventually leads to wheelchair dependency.^6,8^

In order to gain insight into the effects of LOPD on mobility, the current observational study enrolled patients for a 6- to 8-week study in which (a) patients signed-up to the website PatientsLikeMe (PLM, patientslikeme.com) to self-report their disease experience, and (b) a wearable activity tracker (Fitbit One™) was deployed to measure their mobility in a remote setting. The objectives were to capture real-world mobility data through a consumer wearable device in LOPD subjects to: (1) evaluate the willingness to adopt wearable devices for passive and active health monitoring and (2) explore the relationship between patient characteristics and disease experience (symptoms and impact) with device-measured mobility.

## Results

### Patients

Of 40 screened subjects, 5 were ineligible at screening (3 incompletes and 2 screen outs). Of the 35 subjects who completed the baseline survey, the final study cohort comprised of 29 participants who shared Fitbit One™ data. As described in Table 1, the mean age of these 29 participants was 43 years, 90% (*N* = 26) were females, and 93% (*N* = 27) had been diagnosed when they were ≥18 years of age (mean age: 37 years, standard deviation: 12 years). Mean diagnostic delay (period from first symptom onset to diagnosis) was 10 years (standard deviation: 11 years); most participants had their disease onset for more than 10 years (55%). Ambulatory assistance (17%) and breathing assistance (38%) were required for a subset of patients within 7 days prior to baseline. At the time of the study, 86% of participants were treated with ERT (alglucosidase alfa) and one patient (3%) was on an investigational ERT (avalglucosidase alfa).

**Table 1:** Participant demographics and baseline clinical information

Parameter	Final study cohort (*N* = 29)
Age, years	
Mean (SD)	43 (10)
Median (IQR)	41 (35, 53)
20–39	45% (*N* = 13)
40–69	55% (*N* = 16)
Sex, % (*N*)	
Male	10% (*N* = 3)
Female	90% (*N* = 26)
Race, % (*N*)^a^	
White	86% (*N* = 25)
Non-white	14% (*N* = 4)
Age at onset, years	
Mean (SD)	26 (13)
Median (IQR)	30 (17, 35)
<18	21% (*N* = 6)
≥18	59% (*N* = 17)
Unknown	21% (*N* = 6)
Age at diagnosis, years	
Mean (SD)	37 (12)
Median (IQR)	36 (29, 46)
<18	7% (*N* = 2)
≥18	93% (*N* = 27)
Disease duration^b^, years	
Mean (SD)	18 (12)
Median (IQR)	14 (7, 24)
<10	24% (*N* = 7)
≥10	55% (*N* = 16)
Unknown	21% (*N* = 6)
Diagnostic delay^c^, years	
Mean (SD)	10 (11)
Median (IQR)	6 (2, 13)
<10	45% (*N* = 13)
≥10	34% (*N* = 10)
Unknown	21% (*N* = 6)
Mobility (past 7 days), % (*N*)	
Assisted	17% (*N* = 5)
Unassisted	83% (*N* = 24)
Breathing assistance, % (*N*)	
Assisted (BiPAP, CPAP)	38% (*N* = 11; BiPAP = 8, CPAP = 3)
Unassisted	62% (*N* = 17)
Treatments, % (*N*)	
ERT (alglucosidase alfa)	86% (*N* = 25)
Pain medications (OTC)	31% (*N* = 9)
Pain medications (Rx)	14% (*N* = 4)
Walking aid	24% (*N* = 7)
Physiotherapy	3% (*N* = 1)
Investigational ERT (avalglucosidase alfa)	3% (*N* = 1)
Exercise	3% (*N* = 1)
None	7% (*N* = 2)

*BiPAP* bilevel positive airway pressure, *CPAP* continuous positive airway pressure, *ERT* enzyme replacement therapy, *IQR* interquartile range, *OTC* over the counter, *Rx* prescription, *SD* standard deviation

^a^Non-white includes African American (*N* = 1), Native American (*N* = 1), and unknown (*N* = 2)

^b^Disease duration: current age−age at first symptom onset

^c^Diagnostic delay: age at diagnosis−age at first symptom onset

Among the full study cohort (*N* = 29), engagement was high for uploading wearable tracking data but less so with symptom reporting. When frequency (number of days uploaded/number of days in the study) was calculated for activity tracking, 94% of patients uploaded activity data for >50% of the number of study days and 67% uploaded data for >90% of the number of study days (see Table 2). In contrast, only 24%, 3%, and 3% of patients uploaded >90% of the time for daily sleep, the daily InstantMe question, and weekly symptoms, respectively.

**Table 2:** Participant engagement and reporting

	Activity tracking (daily)	Sleep tracking (daily)^b^	InstantMe tracking (daily)	Symptom tracking (weekly)
Mean^a^	88%	60%	49%	27%
Median^a^	98%	69%	55%	14%
Frequency of reporting (% total days)				
>90%	67% (*N* = 20)	24% (*N* = 7)	3% (*N* = 1)	3% (*N* = 1)
75–89%	14% (*N* = 4)	10% (*N* = 3)	7% (*N* = 2)	0% (*N* = 0)
50–74%	10% (*N* = 3)	31% (*N* = 9)	52% (*N* = 15)	0% (*N* = 0)
25–49%	3% (*N* = 1)	17% (*N* = 5)	17% (*N* = 5)	24% *(N* = 7)
0–25%	3% (*N* = 1)	17% (*N* = 5)	21% (*N* = 6)	72% (*N* = 21)
Reported on ≥1 day	100% (*N* = 29)	97% (*N* = 28)	86% (*N* = 25)	52% (*N* = 15)

^a^Values are number of days uploaded/number of days in study for all except symptom tracking, which is number of days uploaded/number of weeks in study. The first week post-baseline (considered the run-in period) was excluded from the denominator. The study period ranged from 36 to 49 days, after excluding the run-in period

^b^One patient did not use the sleep function and was removed from all analyses of sleep data

### Mobility vs. general population and other chronic diseases

Pedometer-based step count data were compared with studies from the general population and patients with other chronic diseases. As shown in Table 3, patients with LOPD were less ambulatory (mean: 3145 steps) than patients in the general population (mean: 5117 steps),^9^ and patients with chronic obstructive pulmonary disease and multiple sclerosis.^10–12^ Note, however, that different activity trackers and methodology were used in each study. A similar observational study using the PLM platform tracked the activity of patients with multiple sclerosis (MS), and these patients also had higher activity (mean: 4393 steps) than the LOPD subjects.^13^

**Table 3:** Mobility (step count) in LOPD subjects relative to the general population and chronic disease patients

Study	Population	Analyses sample	Study design	Wearable device	Descriptive statistics
Free-Living Mobility Assessment Using Wearable Technology in U.S. Adults with Late-Onset Pompe Disease (Current manuscript prepared by Sanofi Genzyme and PatientsLikeMe)	US patients (≥18 years) with late-onset Pompe disease • Age (mean): 43 years • Sex: 90% female • Race: 86% white Excluded patients who were bedridden, required a wheelchair all day, or required invasive ventilation	29 patients	Observational study with continuous remote monitoring physical activity (at home) for 6–8 weeks	Fitbit One™ pedometer (3-axis accelerometer that clips onto an individual’s belt, pocket, or bra)	Mean: 3145 steps per day (range: 722–6304)
Pedometer-Measured Physical Activity and Health Behaviors in United States Adults^9^	US general population (≥18 years) • Age (mean): 46 years • Sex: 54% female • Race: 89% white Only adult cohort with valid data reported for analyses	1136 respondents	Observational study data collected over a 2-day period (at home)	Accusplit AE120 pedometer (worn on the belt or waistband during all waking hours)	Mean: 5117 steps per day
Promoting physical activity in COPD: insights from a randomized trial of a web-based intervention and pedometer use^12^	US Veterans with COPD (≥40 years) • Age (mean): 69 years • Sex: 1.5% female • Race: 92% White Population age ≥40 years with ≥10 pack-year smoking history	109 patients (intervention arm, *n* = 57; control arm, *n* = 52)	Randomized control trial with an intervention arm (web-based intervention plus pedometer) and control arm (pedometer only) for 3 months	Omron HJ-720 ITC pedometer (worn during all waking hours)	Baseline data Mean: 3445 steps per day (intervention arm: 3149; control arm 3770) 7 days baseline data reported as it is more likely to be comparable to free living behavior
Use of pedometer and Internet-mediated walking program in patients with chronic obstructive pulmonary disease^10^	US patients with COPD (≥40 years) • Age (mean): 56 years • Sex: 46% female • Race: 75% white Population age ≥40 years, self-reporting emphysema, asthma, bronchitis and current/former smokers	24 patients	Randomized control trial to test an Internet-mediated walking program for 16 weeks (comparator arm included patients without COPD)	Omron HJ-720 ITC pedometer (worn during all waking hours)	Baseline data Mean: 3429 steps per day (range: 642–7166) 7 days baseline data reported as it is more likely to be comparable to free living behavior
Continuous daily assessment of multiple sclerosis disability using remote step count monitoring^11^	US Patients (≥18 years) with multiple sclerosis • Age (mean): 50 years • Sex: 64% female population included relapsing or progressive type who could walk unaided for ≥2 min	99 patients (38 progressive; 61 relapsing)	Observational study with continuous remote monitoring (at home) for 4 weeks	Fitbit Flex™ pedometer (3-axis accelerometer that was worn as a wrist bracelet on the non-dominant wrist)	Mean: 5478 steps per day (range: 533–18,649)
Free-Living Physical Activity Monitoring in Adult U.S. Patients with Multiple Sclerosis using a Consumer Wearable Device^13^	US patients (≥18 years) with multiple sclerosis • Age (mean): 52 years • Sex: 75% female Excluded patients who required a wheelchair for most of their daily activities Analyses were conducted in patients with complete data on all covariates and at least 7 days of wearable device data	114 patients	Observational study remote monitoring physical activity (at home) for 3–4 weeks	Fitbit One™ pedometer (3-axis accelerometer that clips onto an individual’s belt, pocket, or bra)	Mean: 4393 steps per day

*COPD* chronic obstructive pulmonary disease, *MS* multiple sclerosis

### Mobility by patient characteristics

**Fig. 1:**
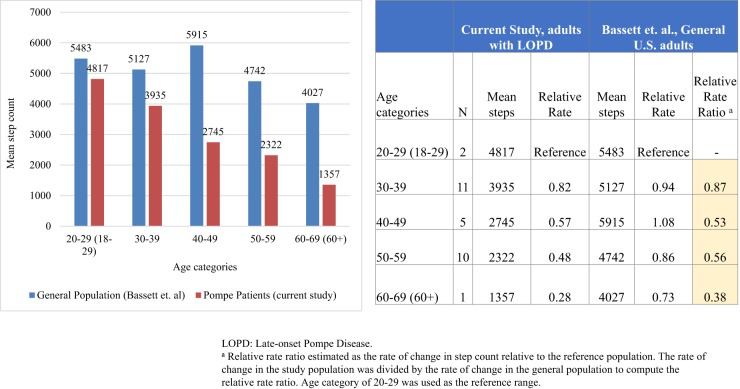
Figure compares difference in mobility by decades in the studied LOPD population relative to the general population. Sample sizes by decades in the current study are small; statistical tests of difference were not performed

Mobility varied by age, diagnostic delays, disease duration, ambulatory status, and “fatigue and pain” score, activity items, among other factors. Although the sample size was small, younger participants averaged more steps than older participants (4071 for 20–39 years vs. 2394 for 40–69 years, *p* < 0.01), with an inverse association between mobility and age which is greater in magnitude compared to the general population (Fig. 1).

Mean step count differed by participant characteristics—diagnostic delay (3584 for <10 years vs. 2232 for ≥10 years, *p* < 0.05), disease duration (4219 for <10 years vs. 2462 for ≥10 years, *p* < 0.05), and ambulatory status (1883 for assisted vs. 3408 for unassisted, *p* < 0.05) (Table 4). Exploratory analyses that tested the interaction between age and diagnostic delay suggest that younger LOPD subjects (20–39 years) have a greater impact from diagnostic delays than patients in the older age group (40–69 years) (not significant, *p* = 0.20). Of note, among seven younger subjects with diagnostic delay of <10 years, the average step count was 4390; two younger subjects with diagnostic delay of ≥10 years averaged 2223 steps. The difference was much lower in magnitude for older subjects; among six older subjects with diagnostic delay of <10 years, the average step count was 2645; eight older subjects with diagnostic delay of ≥10 years averaged 2234 steps (Supplemental Materials).

**Table 4:** Mobility levels by participant characteristics

		Age	Step count^a^		Peak 1ne-minute activity^b^	
	*N*	Mean ± SD	Mean ± SD	*p* value	Mean ± SD	*p* value
Age, years				<0.01		<0.01
20–39	13	33 ± 5	4071 ± 1567		99 ± 13	
40–69	16	51 ± 6	2394 ± 1063		80 ± 18	
Sex, % (*N*)				0.64		0.44
Male	3	43 ± 9	3546 ± 868		96 ± 9	
Female	26	43 ± 11	3099 ± 1607		87 ± 19	
Age at onset, years				0.24		0.83
<18	6	42 ± 11	2337 ± 1136		86 ± 22	
≥18	17	45 ± 9	3229 ± 1682		84 ± 18	
Unknown^d^	6	40 ± 14	3717 ± 1331		101 ± 6	
Age at diagnosis, years				0.90		0.41
<18	2	29 ± 11	3015 ± 666		99 ± 12	
≥18	27	44 ± 10	3155 ± 1594		87 ± 18	
Disease duration^c^, years				<0.05		0.09
<10	7	38 ± 9	4219 ± 1806		95 ± 18	
≥10	16	46 ± 9	2462 ± 1173		81 ± 18	
Unknown^d^	6	40 ± 14	3717 ± 1331		101 ± 6	
Diagnostic delay^e^, years				<0.05		<0.05
<10	13	41 ± 8	3584 ± 1626		92 ± 15	
≥10	10	48 ± 10	2232 ± 1211		76 ± 21	
Unknown^d^	6	40 ± 14	3717 ± 1331		101 ± 6	
Mobility (past 7 days), % (*N*)				<0.05		0.73
Assisted	5	53 ± 11	1883 ± 389		86 ± 17	
Unassisted	24	41 ± 9	3408 ± 1565		89 ± 19	
Breathing assistance, % (*N*)				0.91		0.79
Assisted (BiPAP, CPAP)	11	49 ± 10	3101 ± 1427		89 ± 18	
Unassisted	18	40 ± 9	3173 ± 1648		88 ± 19	
Treatments, % (*N*)^f^						
ERT (alglucosidase alfa)	25	44 ± 10	2975 ± 1405	0.14	86 ± 18	0.11
Pain medications (OTC)	9	45 ± 11	2359 ± 1264	0.06	80 ± 22	0.10
Pain medications (Rx)	4	45 ± 12	1979 ± 1184	0.10	73 ± 23	0.07
Walking aid	7	52 ± 9	1710 ± 811	<0.01	78 ± 24	0.07
Physiotherapy	1	39	4193	NE	97	NE
Investigational ERT (avalglusidase alfa)	1	36	2544	NE	91	NE
Exercise	1	56	3178	NE	94	NE
None^g^	2	34 ± 1	6066 ± 337	<0.01	107 ± 8	0.13

*BiPAP* bilevel positive airway pressure, *CPAP* continuous positive airway pressure, *ERT* enzyme replacement therapy, *OTC* over the counter, *Rx* prescription, *SD* standard deviation, *NE* not estimable; statistical comparison performed using *t*-tests; level of significance set at *α* = 0.05 (two-tailed)

^a^Patient-level median of step count throughout the study period used for analyses

^b^Measured as the highest daily step count within consecutive 1one-min periods; patient-level median of peak 1-min activity throughout the study period used for analyses

^c^Disease duration: current age−age at first symptom onset

^d^Unknown category excluded from statistical comparison

^e^Diagnostic delay: age at diagnosis−age at first symptom onset

^f^Statistical comparison performed for participants on the treatment listed vs. not on the treatment

^g^Two subjects who were not on any treatment were younger (mean age: 34 years) with shorter diagnostic delay (mean: 1 year) and disease duration (mean: 4 years), likely to be newly diagnosed LOPD

Similar findings were observed on testing the interaction between age and disease duration (not significant, *p* = 0.26). Among five younger subjects with disease duration of <10 years, the average step count was 4798; four younger subjects with disease duration of ≥10 years averaged 2797 steps. The difference in magnitude for older subjects was lower; among two older subjects with disease duration of <10 years, the average step count was 2771; 12 older subjects with disease duration of ≥10 years averaged 2349 steps (Supplemental Materials).

With respect to peak 1-min activity; younger participants averaged greater than older participants (99 for 20–39 years vs. 80 for 40–69 years, *p* < 0.01); those with shorter diagnostic delays demonstrated higher activity (92 for <10 years vs. 76 for ≥10 years, *p* < 0.05) (Table 4).

Step count was associated with “fatigue and pain” score as measured by Pompe Disease Symptom Scale (PDSS) (*p* < 0.05); and mobility-related items of the Pompe Disease Impact Scale (PDIS), namely, walking (*p* < 0.01), climbing stairs (<0.05), and squatting (<0.01) (Table 6). Peak 1-min activity of participants was associated with the “fatigue and pain” score of the PDSS scores (*p* < 0.01), walking (<0.05), and squatting (*p* < 0.05) items of the PDIS (<0.05) (Table 5).

**Table 5:** Mobility levels and patient-reported outcomes

		Age	Step count^a^		Peak one-minute activity^b^	
	*N*	Mean ± SD	Mean ± SD^c^	*p* value	Mean ± SD^c^	*p* value
PDSS—“Breathing difficulties”	29	43 ± 10	−0.24 (Pearson’s *r*)	0.21	−0.19 (Pearson’s *r*)	0.32
PDSS—“Fatigue and pain”	29	43 ± 10	−0.42 (Pearson’s *r*)	<0.05	−0.49 (Pearson’s *r*)	<0.01
PDIS—“Mood” score	29	43 ± 10	−0.04 (Pearson’s *r*)	0.86	−0.02 (Pearson’s *r*)	0.92
PDIS—Walking				<0.01		<0.05
Did not walk	0	NA	NA		NA	
Walked with assistance	5	55 ± 2	1542 ± 847		71 ± 26	
Walked without assistance	24	41 ± 10	3480 ± 1447		92 ± 14	
PDIS—Climbing stairs				<0.05		0.06
No	11	48 ± 9	2400 ± 1544		80 ± 21	
Yes	18	41 ± 11	3601 ± 1391		93 ± 15	
PDIS—Rising from a sitting position				NA		NA
No	1	41	4058		106	
Yes	28	44 ± 11	3112 ± 1648		88 ± 18	
PDIS—Bending over				NA		NA
No	1	54	722		43	
Yes	28	43 ± 10	3232 ± 1497		90 ± 16	
PDIS —Squatting				<0.01		0.05
No	17	48 ± 9	2513 ± 1228		82 ± 17	
Yes	12	37 ± 9	4041 ± 1539		97 ± 16	

*PDSS* Pompe Disease Symptom Scale, *PDIS* Pompe Disease Impact Scale, *SD* standard deviation, *NA* not applicable; Statistical comparison performed using *t*-tests; level of significance set at *α* = 0.05 (two-tailed)

^a^Patient-level median of step count throughout the study period used for analyses

^b^Measured as the highest daily step count within consecutive one-min periods

^c^Unless otherwise specified; patient-level median of peak one-minute activity throughout the study period used for analyses

## Discussion

In LOPD, the ability to perform tasks requiring limb and girdle muscle strength such as walking, running, and climbing stairs is progressively impaired, finally leading to dependency on a wheelchair. This ambulation-associated dysfunction may be the presenting symptom, and lower limb muscle strength is thought to continue to decline by approximately 7% per year.^3,7,14^

The current early pilot aimed to evaluate the utility and practical applications of remote activity monitoring LOPD patients using a consumer wearable device. The study revealed three key findings. First, the data demonstrate good compliance with passive remote monitoring in patients with Pompe disease, with 83% of the subjects who completed the baseline assessment uploading wearable data during the study period with a high degree of compliance. More than 80% of these participants uploaded data for ≥75% of the days during a 6- to 8-week study. A total of 59% of the participants would recommend using an activity tracker to other patients with Pompe disease. Participant feedback at the end of the study indicated interest in devices that tracked respiratory function, nutrition, and physical activity. However, it should be noted that compliance was much lower where data capture required participants to actively engage in data entry (e.g., symptom tracking on the website). Importantly, these findings underscore the need for passive physiological data collection tools (sensors and wearable technology) that can minimize the burden for patients.

Second, the study enabled characterization of overall mobility and peak one-minute activity in LOPD patients based on their clinical presentations. For example, participants with LOPD were less active than people in the general population or those with other chronic diseases that are characterized by motor or respiratory dysfunction. Younger subjects were more active than older subjects, and those with shorter diagnostic delay and disease duration were more active, while those requiring ambulatory support were less active. These findings are consistent with clinical evidence in Pompe disease that suggests a longer diagnostic delay and duration of disease in patients lead to progressive loss of function and greater loss of mobility.^15,16^

Studies have highlighted the broad range of symptom presentation in Pompe disease can lead to diagnostic challenges. Patients with LOPD experience diagnostic gaps ranging from 5 years for patients with symptom onset at >12 years of age to 9.3 years for patients ≤12 years of age.^15^ Findings from this study is consistent with the literature. This diagnostic gap may delay the initiation of treatment; untreated disease results in progressive, irreversible damage to skeletal and respiratory muscles. Such damage leads to significant disability, including progressive loss of mobility, respiratory function, and activities of daily living, and premature death.

Our study found LOPD patients with a diagnosis delay of ≥10 years were less active than those who were diagnosed within 10 years of symptom onset (3548 vs. 2232, *p* < 0.05). We also noted a trend between diagnostic delay and mobility that was more pronounced in younger LOPD subjects. Similarly, patients with disease duration of ≥10 years were less active than those who were symptomatic for less than 10 years (4219 vs. 1806, *p* < 0.05), with a trend between disease duration and mobility that was more pronounced in younger LOPD subjects. It should be noted that the interaction effects were not statistically significant and our study was not powered to detect differences in interaction effect. These findings also hint that those who develop the disease earlier have more severe phenotypes.

Third, our study supports the association between disease-specific patient-reported outcomes (PROs) of disease severity (PDSS) and impact (PDIS) with overall mobility and peak one-minute activity. Mobility measures were strongly correlated with “fatigue and pain” score. Patients who were able to carry out physical activities like walking, climbing stairs, and squatting as measured by the PDIS items also demonstrated higher activity measured via the wearable device. This finding is expected and demonstrated the validity of objectively measured mobility in the context of LOPD patients’ subjective experience with symptoms and disease progression. Similarly, PROs that are not conceptually related with mobility (e.g., mood) were not associated with step count or peak one-minute activity, supporting the discriminant validity of activity measures.

As an early pilot, this study creates a framework that will inform future studies of remote health monitoring in Pompe disease. With advancement in technology and integrated data systems, we anticipate that wearable technology linked with patient self-reports will enable better characterization of Pompe patients and aid physicians in clinical decision making. There is a need for better standardization of data to compare and contrast within disease (heterogeneity) as well as in relation to normative data from the general population and other conditions. A recommendation from our study is that in a progressive neuromuscular condition like Pompe disease has a higher age-related rate of decline than healthy cohorts; hence, to measure a significant improvement in any one patient, the trajectory of change must be referenced and calibrated to the disease population (within-disease) and healthy cohort (between-disease). This study is an initial step in that direction. Finally, our results show promising trends to indicate that wearable technology coupled with PROs offer a new approach to evaluate patient-relevant outcomes in both interventional and observational studies. Future studies with longer follow-up periods are required to expand upon this methodology and potentially monitor patients’ health status over time.

The current study has several limitations. First, the study was intended as a pilot with limited data collected on the physical status, normal home and ambulation environment, behaviors, and demographic status of participants. Second, pedometer-based step counting does not characterize all movement disturbances (e.g., gait) in patients with life changing medical conditions. Third, the comparisons with other studies are also potentially confounded by demographic differences, period of observation, behavioral reactivity, study design, and type of device used. Fourth, data were patient-generated only and not combined with clinical assessments. While such data provide valuable insights into the subjective experience in patients, combining objective clinical assessments should be considered in future studies to understand the inter-relationship with clinical markers of disease progression. Fifth, as a pilot study, only descriptive data analyses were possible with some of the stratified and interaction analyses conducted on very small samples. The study was not powered to effectively detect these changes. Multivariable analyses to test the independent association of patient characteristics with mobility were not possible. Lastly, the study sample is skewed towards females, and a lower proportion of patients required breathing and ambulatory assistance and may not be generalizable to the LOPD population at large.

Data from this pilot study enabled characterization of LOPD patients based on their mobility. Measured mobility outcomes conformed to clinical presentations and were higher in participants who were younger, had shorter diagnostic delays, had shorter disease duration, were not on ambulatory assistance; correlations were observed with patient-reported “fatigue and pain” and physical activity items. The findings suggest that passive remote monitoring using wearable technology may yield valuable real-world observational data on mobility in LOPD patients.

## Methods

### Study population

Inclusion criteria were self-reported diagnosis of LOPD, age ≥ 18 years and US resident. Exclusion criteria were those who were bedridden or required a wheelchair all day, and those who required invasive ventilation. Participants were recruited from the Acid Maltase Deficiency Association (AMDA, *N* = 28) and from the existing membership of PLM (*N* = 1). AMDA is a patient organization that promotes public awareness of Pompe disease. PLM is an online research platform, designed to allow platform members to share data about their conditions, treatments, symptoms, and comorbidities through structured data collection, but with some features of an online social network.^17^

### Study design and assessments

**Fig. 2:**
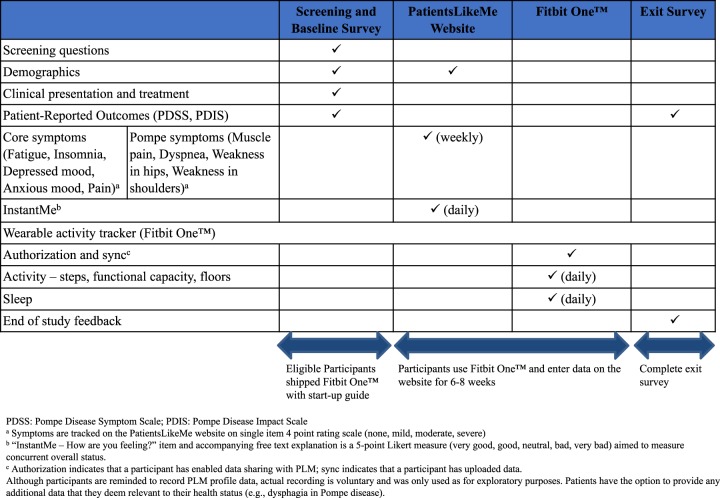
Figure shows the data collection schema and schedule of assessments during the study

As shown in Fig. 2, after a screening and baseline assessment, data were captured over 6 to 8 weeks through a wearable activity tracker (Fitbit One^TM^) (daily) and self-assessment (weekly and daily) via the PLM website, with an exit survey at the conclusion of the study.

### Ethics approval and consent to participate

Informed consent was obtained from participants electronically. A statement of research information was also sent to participants when they received their wearable devices by postal mail. Independent ethics review was sought and the study protocol received ethical approval from the New England Institutional Review Board (NEIRB) [Study Number: 120160856; Approval date: 10 August 2016].

### Demographics and patient characteristics measure

Baseline assessment included age, gender, race, age at first symptom onset, age at diagnosis, specialty of diagnosing physician, use of mobility aid (assisted vs. unassisted), use of breathing assistance [oxygen, bilevel positive airway pressure, continuous positive airway pressure], and treatments including prescriptions and over-the-counter. Disease duration was computed as the difference between age and age at first symptom onset. Diagnostic delay was computed as the difference between age at diagnosis and age at first symptom onset.

### Pompe Disease Symptom Scale

The PDSS is a newly developed 12-item patient-reported outcome designed to capture the range and severity of disease-related symptoms experienced by Pompe disease patients. The content validity of the instrument was established via concept elicitation patient interviews and cognitive debriefing. It measures severity of breathing difficulties, fatigue and tiredness, muscle weakness and ache, pain, and headache from the LOPD patient’s perspective.^18^ The items ask patients to rate their worst symptom severity over the previous 24 h using 0–10 numeric rating scales. Principal components analysis was conducted to organize the items of the PDSS scale into subdomains. The PDSS items conformed to a 2 factor solution: (1) a “fatigue and pain” domain and (2) a “breathing difficulties” domain. The “fatigue and pain” domain comprises eight items (tiredness, fatigue, muscle weakness anywhere, muscle weakness in lower body, muscle weakness in arms, muscle weakness in upper body, muscle aches, pain). The “breathing difficulties” domain comprises two items (breathing difficulty, breathing difficulty lying down). Two items did not fit the factor solution and were not scored (muscle weakness in hand grip, morning headache). PDSS domain scores were calculated by summing the domain level scores; the possible ranges of scores are as follows: “fatigue and pain” domain (0–80), “breathing difficulties” (0–20). Patients completed the PDSS at baseline and at the exit survey; the baseline survey data are used here (Table 6).

**Table 6:** Items and domains of the PDSS and PDIS

Items	Response option	Domain	Score range
PDSS			
Breathing difficulties	0–10	Breathing difficulties	0–20
Breathing difficulties lying down	0–10	Fatigue and pain	0–80
Tiredness	0–10		
Fatigue	0–10		
Muscle weakness anywhere	0–10		
Muscle weakness in lower body	0–10		
Muscle weakness in arms	0–10		
Muscle weakness in upper body	0–10		
Muscle aches	0–10		
Pain	0–10		
Muscle weakness in hand grip^a^	0–10	Hand grip	Not scored
Morning headache^a^	0–10	Morning headache	Not scored
PDIS			
Anxiety	0–10	Mood	0–30
Worry	0–10		
Depression	0–10		
Walking	Without assistance/with assistance/no	Walking	Categorical
Climbing stairs	yes/no	Climbing stairs	Categorical
Rising from a sitting position	yes/no	Rising from sitting	Categorical
Bending over	yes/no	Bending over	Categorical
Squatting	yes/no	Squatting	Categorical
Exercise^a^	yes/no	Exercise	Not scored
Difficulty walking^a^	0–10	Difficulty performing activities	Not scored
Difficulty climbing stairs^a^	0–10		
Difficulty rising from a sitting position^a^	0–10		
Difficulty bending over^a^	0–10		
Difficulty squatting^a^	0–10		
Difficulty tolerating exercise^a^	0–10	Difficulty tolerating exercise	Not scored

^a^The item is not included in scoring

### Pompe Disease Impact Scale

The PDIS is a newly developed 15-item patient-reported outcome designed to capture the impact of LOPD on mood (depression, worry, anxiety) and mobility-related physical activities (walking, climbing stairs, rising from a sitting position, bending over, squatting, and exercise) in the past 24 h.^18^ Some items ask about the severity of a particular impact on a 0–10 scale (depression, worry, anxiety), while other items ask whether or not the patient could complete each mobility-related activity in the past 24 h and the level of difficulty associated with the activities that were completed (0–10 scale). Inter-item correlations suggested a “mood” domain (anxiety, worry, depression; range 0–30). Missing data for the mobility-related activity items in the PDIS (due to skip patterns when patients reported that they did not complete an activity) and small sample size prohibited completion of further psychometric analysis. These relevant mobility-related activities (walking, climbing stairs, rising from a sitting position, bending over, squatting) were therefore analyzed at the item-level. Patients completed the PDIS at baseline and at the exit survey; the baseline survey data are used here (Table 6).

### Mobility

Mobility was tracked using the Fitbit One™ activity tracker (manufactured by Fitbit, San Francisco, CA), a wearable device that clips onto an individual’s belt, pocket, or bra.^19^ Daily activity is measured via a three-axis accelerometer that turns movement into digital data when attached to the body. Categories of data counted by the tracker include steps, floors, calories burned, distance, and sleep. The device uses an algorithm that detects motion patterns indicative of walking, with a motion size above a threshold counted as a step. A sensor calculates altitude based on atmospheric pressure, allowing determination of floors (i.e., climbing steps up to the next floor of a building). When the sleep mode is turned on, total hours of sleep and the number of awakenings are measured. A systematic review of wearable activity trackers showed that the Fitbit One™ has high validity and reliability for steps.^20^ The device has a battery life of 14 days and allows wireless data upload (synchronization). The participants uploaded data daily to the PLM website after an initial authorization. While individualized messages reminded the participants to upload Fitbit One™ data, because uploading was done on a voluntary basis, some missing data were observed. Data on the following were measured and analyzed: steps, as an overall assessment of mobility; peak daily activity, which was the maximum step count in one-minute period; floors, which was an increase in altitude by 10 feet accompanied by a detection of steps and forward motion; and sleep, in terms of total sleep duration and number of awakenings.

### Website assessments

General symptoms (fatigue, insomnia, depressed mood, anxious mood, pain) and Pompe-specific symptoms (muscle pain, dyspnea, weakness in hips, weakness in shoulders) on the website were rated by participants weekly on a 4-point scale (none, mild, moderate, or severe). Participants also answered a daily “InstantMe” question (How are you feeling?) using the following ratings: very good, good, neutral, bad, or very bad. Website assessments were exploratory and the analyses were not in scope for this paper.

### Exit survey

At the end of the study, a subset of the subjects responded to an exit survey regarding user experience and opinions about disease management/self-monitoring.

### Data analyses

Data from Fitbit One™ were extracted via the manufacturers application program interface (API). Initial reviews were performed to examine data density, outliers, and unreasonable values. First, day-level data were obtained for all participants. Missing day-level data and daily value <100 steps (likely attributable to low wear time) were removed from analyses. Data from the first recorded day were removed as these data points may be reflective of a partial day (e.g., participant connects a device the first time on the second half of the day). Second, patient-level median values were computed for each outcome from all available days in the study (adherence was high and all patient had at least 7 days of data). Patient-level median steps were used as a proxy for overall mobility; median of maximum step count in a one-minute period was used as a measure of peak one-minute activity. Floor and sleep data were analyzed but are out of scope for the present paper.

Descriptive statistics (mean, standard deviation, median, quartiles, frequency, and percentage) were computed on baseline patient demographic and clinical characteristics. Stratified analyses were conducted to compare mobility levels in participants by demographic, clinical characteristics, and patient-reported outcomes (disease severity and impact). Statistical tests of significance between strata were tested using *t*-tests. Pearson’s correlation coefficients (*r*) were computed where applicable. Finally, while not directly comparable, mobility was compared between our study population and other general and chronic disease population-based estimates from publicly available data sources. All tests were two-tailed and the level of significance was set at *α* = 0.05.

### Reporting summary

Further information on experimental design is available in the Nature Research Reporting Summary linked to this article.

## Supplementary information

Supplemental Materials

Reporting Summary Checklist

## Data Availability

Qualified researchers may request access to patient-level data, code and related study documents including the clinical study report, study protocol with any amendments, blank case report form, statistical analysis plan, and dataset specifications. Patient-level data will be anonymized and study documents will be redacted to protect the privacy of study participants. Further information on Sanofi’s data sharing criteria, eligible studies, and process for requesting access can be found at https://www.clinicalstudydatarequest.com.

## Supplementary information

**Supplementary information** accompanies the paper on the *npj Digital Medicine* website (10.1038/s41746-019-0143-8).

## Code Availability

Data management was performed in R 3.5.1; analyses were conducted using SAS 9.4 and Microsoft Excel 2010. Code may be available upon reasonable request.

## References

[1] Angelini C (2015). Spectrum of metabolic myopathies. Biochim. Biophys. Acta.

[2] Kishnani PS (2013). Timing of diagnosis of patients with Pompe disease: data from the Pompe registry. Am. J. Med. Genet..

[3] Al-Lozi, M. T., Amato, A. A., Barohn, R. J., Cupler, E. J., Kishnani, P. S., Leshner, R. T. & Mozaffar, T. Diagnostic criteria for late-onset (childhood and adult) Pompe disease. *Muscle Nerve* **40**, 149–160 (2009). 10.1002/mus.21393 19533647

[4] Winkel LP (2005). The natural course of non-classic Pompe’s disease; a review of 225 published cases. J. Neurol..

[5] Reuser, A. J., Hirschhorn, R. & Kroos, M. A. Pompe Disease: Glycogen Storage Disease Type II, Acid α-Glucosidase (Acid Maltase) Deficiency. In *The Online Metabolic and Molecular Bases of Inherited Disease* (eds Valle, D., Beaudet, A. L., Vogelstein, B., Kinzler, K. W., Antonarakis, S. E., Ballabio, A., Gibson, K. & Mitchell, G.). (McGraw-Hill, New York, NY, 2014).

[6] Hagemans ML (2005). Clinical manifestation and natural course of late-onset Pompe’s disease in 54 Dutch patients. Brain.

[7] van der Beek NA (2012). Clinical features and predictors for disease natural progression in adults with Pompe disease: a nationwide prospective observational study. Orphanet J. Rare Dis..

[8] van der Beek NA (2009). Rate of disease progression during long-term follow-up of patients with late-onset Pompe disease. Neuromuscul. Disord..

[9] Bassett DR, Wyatt HR, Thompson H, Peters JC, Hill JO (2010). Pedometer-measured physical activity and health behaviors in U.S. adults. Med. Sci. Sports Exerc..

[10] Moy ML (2010). Use of pedometer and Internet-mediated walking program in patients with chronic obstructive pulmonary disease. J. Rehabil. Res. Dev..

[11] Block VJ (2017). Continuous daily assessment of multiple sclerosis disability using remote step count monitoring. J. Neurol..

[12] Wan ES (2017). Promoting physical activity in COPD: insights from a randomized trial of a web-based intervention and pedometer use. Respir. Med..

[13] DasMahapatra, P., Chjauzzi, E., Bhalerao, R. & Rhodes, J. Free-living physical activity monitoring in adult U.S. patients with multiple sclerosis using a consumer wearable device. *Digital Biomark* **2** (2018). 10.1159/000488040 PMC7015360 32095756

[14] Wokke JH (2008). Clinical features of late-onset Pompe disease: a prospective cohort study. Muscle Nerve.

[15] Genzyme. Pompe Registry (2015). In *Global Annual Regulatory Report. Covers the Period of 05 July 2014 to 03 July 2015. Myozyme(R) (algluocosidase alfa)*. Cambridge, MA (2015).

[16] Hagemans ML (2005). Disease severity in children and adults with Pompe disease related to age and disease duration. Neurology.

[17] Brownstein CA, Brownstein JS, Williams DS, Wicks P, Heywood JA (2009). The power of social networking in medicine. Nat. Biotechnol..

[18] Hamed, A. et al. PRO instrument development for late-onset Pompe disese. In *13th Annual WORLD Symposium*, 13–17, 2017, San Diego, CA.

[19] Fitbit Inc. *Fitbit One User Manual* (2017). https://www.fitbit.com/one#features.

[20] Evenson KR, Goto MM, Furberg RD (2015). Systematic review of the validity and reliability of consumer-wearable activity trackers. Int. J. Behav. Nutr. Phys. Act..

