# Supplementary material for: Mobility assessment using wearable technology in patients with late-onset Pompe disease
Source: NPJ Digit Med. 2019 Jul 22;2:70. doi: 10.1038/s41746-019-0143-8 (PMC6646308; doi:10.1038/s41746-019-0143-8)
Supplement: Supplementary file 1 — Supplemental Materials [file 41746_2019_143_MOESM1_ESM.pdf]

Appendix 1. Difference in Mobility by Age and Diagnostic Delay

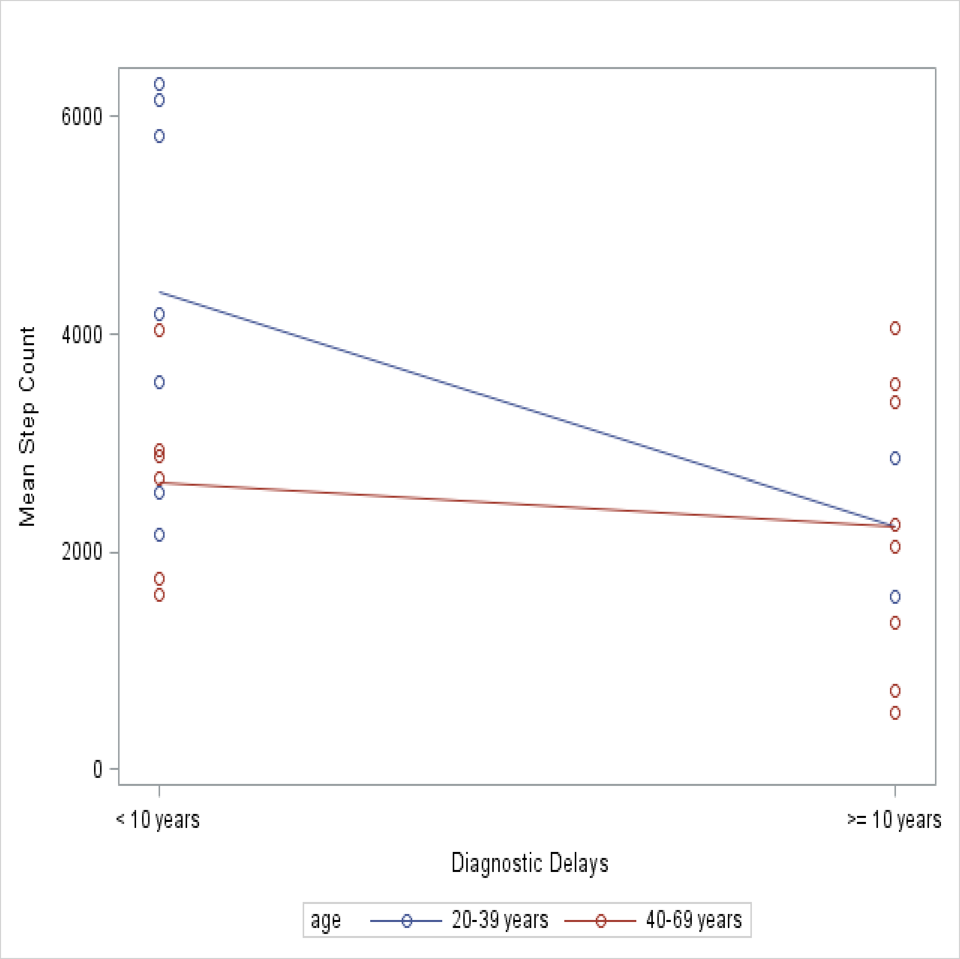

Figure compares difference in physical activity by age and diagnostic delays. The magnitude of difference in activity by diagnostic delay is greater in younger LOPD subjects (20-39 years) relative to older subjects (40-69 years).

Test for Interaction between Age and Diagnostic Delay: (F value=1.78, p=0.20)

Appendix 2. Difference in Mobility by Age and Disease Duration

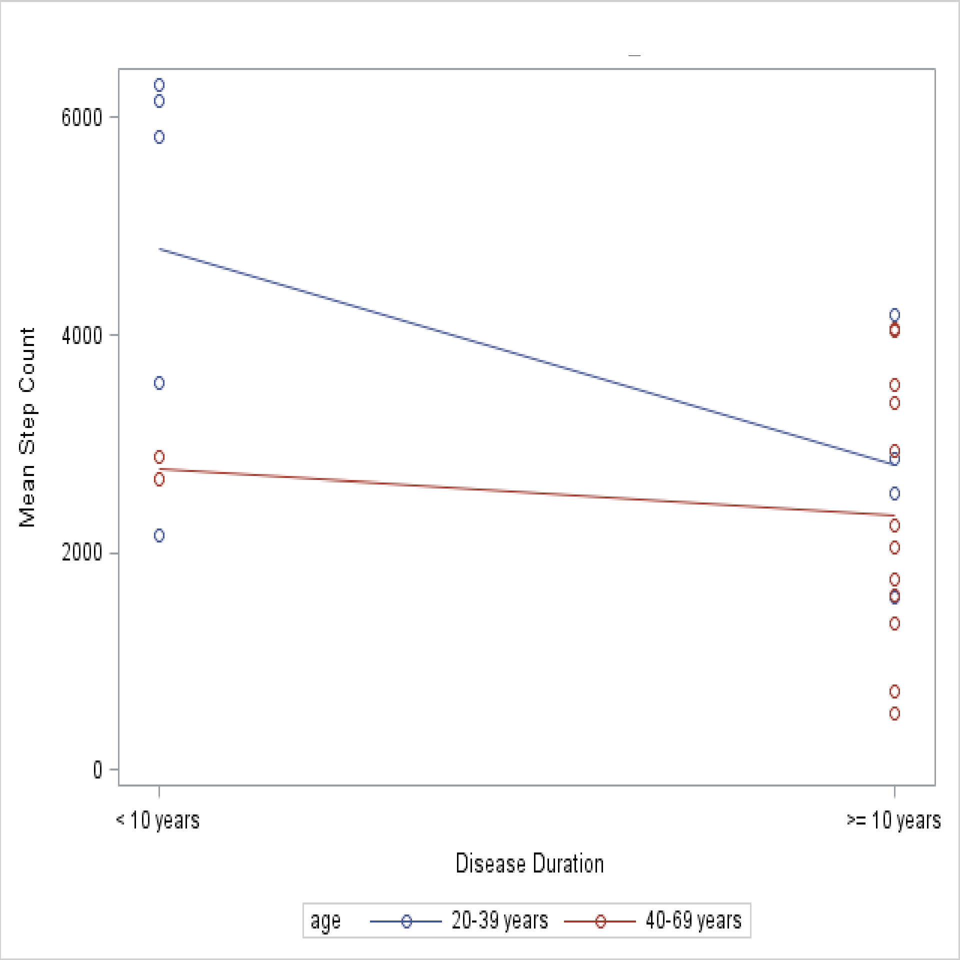

Figure compares difference in physical activity by age and disease duration. The magnitude of difference in activity by disease duration is greater in younger LOPD subjects (20-39 years) relative to older subjects (40-69 years).

Test for Interaction between Age and Disease Duration: (F value=1.36, p=0.26)
